# A Novel Type of Coracobrachialis Muscle Variation and a Proposed New Classification

**DOI:** 10.7759/cureus.1466

**Published:** 2017-07-13

**Authors:** Georgi P Georgiev, Boycho Landzhov, R. Shane Tubbs

**Affiliations:** 1 Department of Orthopedics and Traumatology, Medical University of Sofia, Bulgaria, University Hospital Queen Giovanna; 2 Department of Anatomy, Histology and Embryology, Medical University of Sofia, Bulgaria, University Hospital Queen Giovanna; 3 Neurosurgery, Seattle Science Foundation

**Keywords:** coracobrachial variations, coracoepitrochlearis muscle, classification

## Abstract

During a routine anatomical dissection in the right brachium of a 75-year-old male cadaver, a novel variation of the coracobrachialis muscle (CB) was discovered. It consisted of three parts: proximal, starting from the upper border of the scapula; medial, presenting the well-accessed CB; and distal, formed by a proximal tendinous part that arose from the coracoid process and then transitioned into a well-defined muscle body and a distal tendinous part attached to the medial epicondyle. We also present a new systematic classification of CB variations, dividing them into two simple groups.

## Introduction

The different anatomical variations of the coracobrachialis muscle (CB) have seldom been described in the literature [1-3]. The numerous terms used to describe them have confused anatomists and surgeons alike. Moreover, because they are rare, such variations have not been well-documented [4].

In most species, the CB has three portions: the CB longus, the CB medius, and the CB brevis. In humans, the medius and the longus fuse to form the CB; the brevis is a rarely present third head of the muscle. The musculocutaneous nerve innervates the CB [5].

The CB serves as a guide for locating the axillary artery during a blockade of the brachial plexus [6]. It can also be used as a graft in reconstruction after a mastectomy and in axillary and infraclavicular deformities [2]. Moreover, it can be used for the transfer of vascularized muscle in the treatment of facial palsy [4].

In this case report, we present a novel variation of the CB, and we briefly review the reported variations of the CB and divide them into two simple groups.

## Case presentation

**Figure 1:**
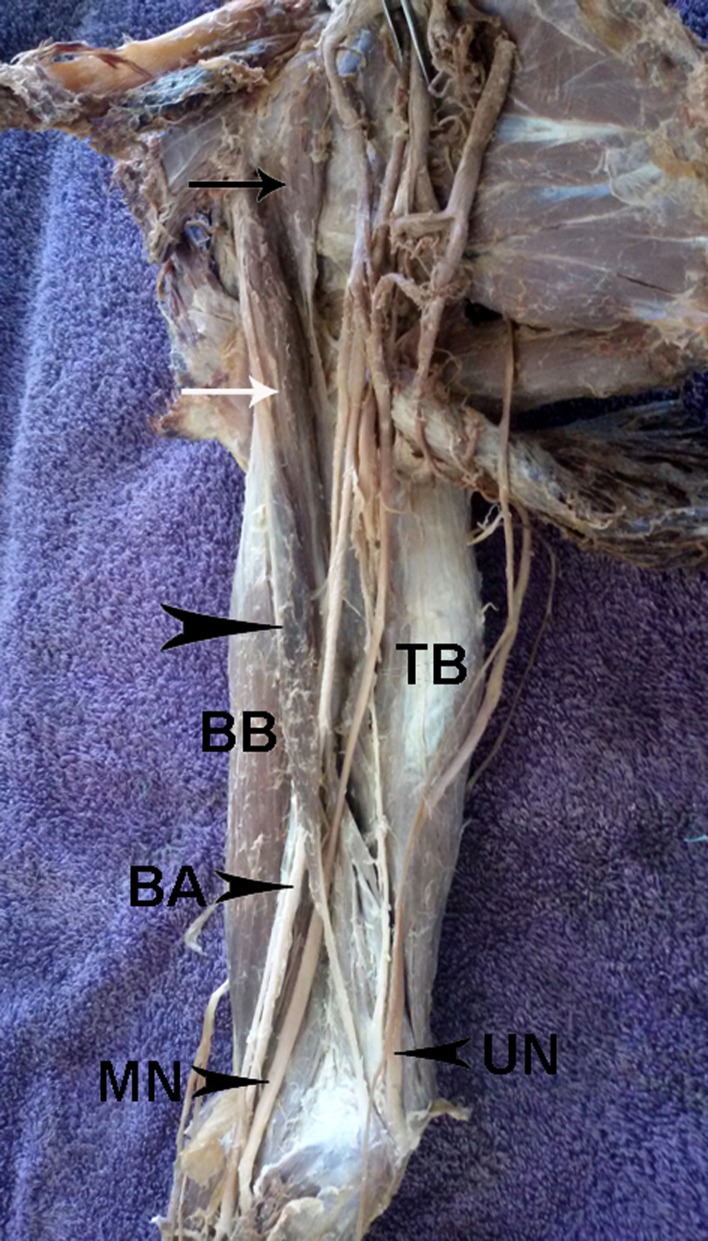
Photograph of the right brachium Muscles – coracoepitrochlearis muscle (arrowhead); classical CB (white arrow); CB brevis or scapulobrachial muscle (black arrow); BB: biceps brachii; TB: triceps brachii Nerves - UN: ulnar nerve; MN: median nerve Artery – BA: brachial artery

A rare case of CB variation was observed during a routine anatomical dissection in the right brachium of a 75-year-old Caucasian male cadaver. The variant CB consisted of three parts. The first started from the superior border of the scapula over the scapular notch and had a well-presented muscular body (length – 130 mm, width – 112 mm) that continued into a slim tendon inserted into the upper third of the medial part of the medial intramuscular septum. The second part corresponded to the classical description of the CB. The most interesting aspect was the aberrant third part, consisting of well-defined proximal and distal tendinous portions and an intermediate muscle belly. The proximal tendinous portion (length – 150 mm, width – 8 mm) originated from the coracoid process together with the CB, continuing into the intermediate muscle belly (length – 28 mm, width – 18 mm), and then into the well-formed distal tendinous portion (length – 80 mm, maximal width – 60 mm), which was attached to the medial epicondyle of the humerus (Figure 1).

From a clinical point of view, the most intriguing portion was the intermediate muscle belly, which passed over the brachial artery and the median nerve in the brachium. The variant muscle was innervated by the musculocutaneous nerve. No medical or surgical history of the cadaver was available. As a cadaveric examination, the present study did not require approval by an ethics committee at our institution, and the work was performed in accordance with the requirements of the Declaration of Helsinki (64th WMA General Assembly, Fortaleza, Brazil, October 2013).

## Discussion

In 1867, Wood first observed that the CB had three parts: the upper part started from the coracoid process and attached to the capsule of the shoulder joint (CB superior, brevis, or rotator humeri); the middle inserted into the mid-part of the humerus and observed in humans (CB proprius or medius); the lower attached to the internal condyloid ridge, the internal intermuscular septum, or the trochlea (CB longus) [3]. Kyou-Jouffroy et al. (as cited by Catli et al.) gave a similar description [7]. In contrast, Mori (1964) described the CB as divided into superficial and deep layers in 16 percent and incompletely divided in 8 percent [8] of Japanese.

Different variations of the CB have been reported mainly in the old anatomical literature (Table 1).

**Table 1:** temp

Variations and terms	Reference
Proximal accessory bands to the lesser tubercle or surgical neck of the humerus (coracobrachialis superior or brevis)	Bergman et al. [1], Wood [3], Bauones and Moraux [4]
Proximal accessory bands started from the conoid ligament of the clavicle and inserted to the medial intermuscular septum	Chouke [5]
Proximal accessory bands to capsule of the shoulder joint (coracocapsularis)	Bergman et al. [1], Wood [3]
Proximal accessory bands to the pectoralis major tendon (coracobrachialis minor s. secundus)	Bergman et al. [1], Wood [3]
Proximal accessory bands to the tendon of latissimus dorsi (coracobrachialis brevis s. rotator humeri, le court coracobrachialis, minor coracobrachial muscle of Cruveilhier)	Bergman et al. [1]
The medial head of the triceps	El-Naggar and Zahir [2]
The brachial fascia	Ilayperuma et al. [6]
Distal insertion to the medial supracondylar ridge (coracobrachialis inferior or longus).	Bergman et al. [1], Wood [3]
Distal insertion to medial intermuscular septum or supracondylar process (coracobrachialis inferior or longus).	Bergman et al. [1], Wood [3]
Distal insertion to the medial epicondyle of the humerus or the antebrachial fascia. (coracobrachialis inferior or longus)	El-Naggar and Zahir [2]

Our review of this literature and the PubMed database did not reveal a similar three-part CB muscle.

Most of the terms used to describe such variants have relied on either the attachment sites of the muscle or its functional characteristics. The reported superior part of our case could be termed the scapulo-brachial muscle or CB brevis. The CB variation inserted into the medial epicondyle of the humerus is usually described as CB inferior or longus. We propose “coracoepitrochlearis muscle” as a more appropriate term for describing the proximal and distal insertions of this muscle, as presented in our case. To simplify the reported variations and terms, we suggest dividing them into two simple groups: (a) CB brevis, inserted into the proximal part of the humerus, and (b) CB longus, inserted into the distal brachium. As a result, the accessory superior part documented in our case can be accepted as CB brevis and be assigned to the first group. The lower part, the coracoepitrochlearis muscle, belongs to the second group when this classification is used. It is evident that such a grouping will simplify the variations in cases presented in the future. We do not use the term CB medius and accept this as the classically described main CB.

It is widely accepted that the CB is of little importance for upper limb function [6]. According to Wood, its upper part contributes to the external rotation of the humerus, while its middle and lower parts ensure the adduction and elevation of the shoulder. Bassett et al. [9] revealed its role as a flexor of the shoulder and its action as a stabilizer of the joint against anterior dislocation. In addition, its proximal insertion around the short head of the biceps brachii could synergize its function in some cases [6].

During embryogenesis, the intrinsic muscles of the arm differentiate from the limb bud mesenchyme of the lateral plate mesoderm. The fusion of these primary muscles generates a single muscle, which then regresses as the layers of the arm muscle differentiate. The presence of an accessory CB could be explained as resulting from a premature termination of this regression process [2].

Although there are currently no clinical reports describing the possible complications arising from such a variant causing nerve and vascular compression because the muscle crosses over the neuro-vascular structures, it could, in principle, cause compression symptoms, as seen in other, reported muscular variations, such as the Struthers ligament, and in the presence of the chondroepitrochlearis muscle, which are well known in the surgical literature [2]. Clinical reports could be lacking first because this variation is rare so there is little information, and second because the limited skin incision performed during a decompression surgery cannot expose the entire variation so it cannot clearly define it. From a surgical point of view, it is important when verifying the variant anatomical structure to resect it without dissection while decompressing the neurovascular bundle.

The coracoepitrochlearis muscle reported here could also cause a confusion when different imaging modalities are used, and it could restrict the abduction of the arm [4]. However, it could serve as an ideal tendinous graft donor, if present, because its excision should not impede the function of the extremity. Mestdagh et al. [10] reported a subcoracoid impingement caused by an accessory CB treated by simple resection.

## Conclusions

In conclusion, knowledge of the variation presented extends the anatomical and clinical literature. This anatomical variant could prove clinically important during traumatic reconstruction in this region, during different esthetic surgical procedures, and for vascularized muscle transfer in the treatment of facial palsy. The potential existence of this and other anomalous muscles should also be considered in the differential diagnosis of neurovascular compression syndromes.

## References

[1] Bergman RA, Afifi AK, Miyauchi R (2017). Illustrated encyclopedia of human anatomic variations. Anatomy Atlas.

[2] El-Naggar MM, Al-Saggaf S (2004). Variant of the coracobrachialis muscle with a tunnel for the median nerve and brachial artery. Clin Anat.

[3] Wood J (1867). On human muscular variations and their relation to comparative anatomy. J Anat Physiol.

[4] Bauones S, Moraux A (2015). The accessory coracobrachialis muscle: ultrasound and MR features. Skeletal Radiol.

[5] Chouke KS (1924). Variation of the coracobrachialis muscle. Anat Rec.

[6] Ilayperuma I, Nanayakkara BG, Hasan R (2016). Coracobrachialis muscle: morphology, morphometry and gender differences. Surg Radiol Anat.

[7] Catli MM, Ozsoy U, Kaya Y (2012). Four-headed biceps brachii, three-headed coracobrachialis muscles associated with arterial and nervous anomalies in the upper limb. Anat Cell Biol.

[8] Mori M (1964). Statistics on the musculature of the Japanese. Okajimas Folia Anat Jpn.

[9] Bassett RW, Browne AO, Morrey BF (1990). Glenohumeral muscle force and moment mechanics in a position of shoulder instability. J Biomech.

[10] Mestdagh H, Maynou C, Cassagnaud X (2002). Accessory coracobrachialis muscle as a cause of anterior impingement syndrome of the rotator cuff in an athlete. Eur J Orthop Surg Traumatol.

